# A Comment on ‘Screening for Obstructive Sleep Apnea Before Coronary Angiography’

**DOI:** 10.1111/crj.70077

**Published:** 2025-04-13

**Authors:** Baris Demirkol, Celal Satici

**Affiliations:** ^1^ Department of Pulmonology, University of Health Sciences Basaksehir Cam and Sakura City Hospital Istanbul Turkey; ^2^ Department of Pulmonology, University of Health Sciences Yedikule Chest Diseases and Thoracic Surgery Training and Research Hospital Istanbul Turkey

**Keywords:** atherosclerosis, coronary angiography, coronary heart disease, endothelial dysfunction, obstructive sleep apnea

Obstructive sleep apnea (OSA) is closely associated with cardiovascular diseases, and its impact on coronary heart disease (CHD) has been increasingly investigated [1, 2]. The pathophysiological mechanisms of OSA, such as recurrent nocturnal hypoxemia and increased sympathetic nervous system activation, can lead to endothelial dysfunction and early atherosclerotic changes, thereby heightening the risk of CHD [2, 3, 4]. Additionally, OSA shares common characteristics with other major cardiovascular risk factors, including hypertension, obesity, and metabolic syndrome, further underscoring the strong relationship between these conditions [5, 6]. Therefore, effective screening and management of OSA may play a significant role in the prevention and treatment of CHD.

We have reviewed with great interest the study by Guo Pei et al., titled “Screening for Obstructive Sleep Apnea Before Coronary Angiography,” which highlights the importance of OSA screening prior to coronary angiography [7]. While this valuable study contributes to the literature, we believe it could yield more impactful results with some refinements.

First, since the primary focus of the study is the importance of OSA screening, selecting OSA rather than CHD as the primary endpoint might be more appropriate. This adjustment could better align the study with its focus on assessing the clinical implications of OSA and provide a more coherent framework for its objectives. Presenting and comparing the characteristics of OSA (+) and OSA (−) groups, along with conducting a multivariable analysis of these data, could also enhance the clarity and interpretability of the findings. Logistic regression analyses based on these groups could more effectively evaluate the independent predictors of OSA detection.

The current multivariable analysis model includes parameters with high collinearity (e.g., total cholesterol, LDL, HDL), which may adversely affect the model's accuracy and result in multicollinearity issues. Evaluating the model's fit using measures such as the Hosmer–Lemeshow test and considering alternative models could help better elucidate the independent effects of each parameter.

Lastly, performing a receiver operating characteristic (ROC) analysis to clarify the relationship between OSA screening and Gensini scores could provide a more precise determination of a cut‐off value with high sensitivity for OSA. This additional analysis would facilitate a more practical interpretation of the results and enhance the study's clinical applicability.

## Author Contributions

Baris Demirkol and Celal Satici contributed to the study's design and implementation, the evaluation and analysis of the results, and the drafting of the manuscript.

## Conflicts of Interest

The authors declare no conflicts of interest.

## Data Availability

Data sharing is not applicable to this article as no datasets were generated or analysed during the current study.
